# MENTAL AND PHYSICAL HEALTH PATHWAYS LINKING EXPOSURE TO HIGHWAYS AND COGNITIVE HEALTH AMONG OLDER MEXICAN AMERICANS

**DOI:** 10.1093/geroni/igae098.0483

**Published:** 2024-12-31

**Authors:** Sadaf Milani, Erika Meza, Carmen Carrion, Helen Meier, Laura Zahodne

**Affiliations:** The University of Texas Medical Branch, Galveston, Texas, United States; Harvard University, Cambridge, Massachusetts, United States; Yale University, New Haven, Connecticut, United States; Institute for Social Research, Institute for Social Research, Michigan, United States; University of Michigan, Ann Arbor, Michigan, United States

## Abstract

Residing in an area with more major roadways is associated with worse cognitive function, yet few studies examine this relationship among older Mexican Americans. Our objective is to explore mental and physical health pathways linking exposure to highways to cognitive performance/function among Mexican Americans aged 75 and older. We used data from the Hispanic Established Population for the Epidemiological Study of the Elderly (2004/2005). Our exposure was the number of primary and secondary roads in a participant’s census tract. Our outcome was the Mini-Mental State Examination (MMSE) score. We examined depressive symptoms (Center for Epidemiologic Studies Depression Scale), neighborhood satisfaction (very satisfied/satisfied vs. neutral/dissatisfied/very dissatisfied), basic activity of daily living (ADL) limitations (7-item modified Katz ADL scale), and physical function (Short Physical Performance Battery) as separate potential mediators, using path analysis. Covariates included age, sex, education, marital status, nativity, language of interview, length of time in residence, and census tract characteristics (urbanicity, affluence, disadvantage, and proportion of Hispanic residents). Our analytic sample (n=1,723) had an average age of 81.5 years (standard deviation: 4.9) and was 61.2% female. Residing in census tracts with more highways was associated with lower MMSE scores. Depressive symptoms, neighborhood satisfaction, and physical function were not mediators while ADL limitations mediated 40% of the relationship between living in census tracts with more highways and MMSE scores. Future work is needed to understand the direct and indirect mechanisms underlying the association between exposure to highways and ADL limitations, as well as between ADL limitations and cognitive health.

